# *Crotonis Fructus* and Its Constituent, Croton Oil, Stimulate Lipolysis in OP9 Adipocytes

**DOI:** 10.1155/2014/780385

**Published:** 2014-11-11

**Authors:** Mi-Seong Kim, Ha-Rim Kim, Hong-Seob So, Young-Rae Lee, Hyoung-Chul Moon, Do-Gon Ryu, Sei-Hoon Yang, Guem-San Lee, Je-Ho Song, Kang-Beom Kwon

**Affiliations:** ^1^Center for Metabolic Function Regulation, Wonkwang University School of Medicine, No. 460 Iksan-Daero, Iksan, Jeonbuk 570-749, Republic of Korea; ^2^BK21 Plus Program and Department of Smart Life-Care Convergence, Graduate School, Wonkwang University, No. 460 Iksan-Daero, Iksan, Jeonbuk 570-749, Republic of Korea; ^3^Department of Oral Biochemistry, Wonkwang University School of Dentistry, No. 460 Iksan-Daero, Iksan, Jeonbuk 570-749, Republic of Korea; ^4^Institute of Customized Physical Therapy, No. 217 Wallgye-ro, Gwangju Metropolitan City 506-303, Republic of Korea; ^5^Department of Korean Physiology, Wonkwang University School of Korean Medicine, No. 460 Iksan-Daero, Iksan, Jeonbuk 570-749, Republic of Korea; ^6^Department of Internal Medicine, Wonkwang University School of Medicine, No. 460 Iksan-Daero, Iksan, Jeonbuk 570-749, Republic of Korea; ^7^Department of Herbology, Wonkwang University School of Korean Medicine, No. 460 Iksan-Daero, Iksan, Jeonbuk 570-749, Republic of Korea; ^8^Department of Sports Industry & Welfare, Wonkwang University School of Natural Science, No. 460 Iksan-Daero, Iksan, Jeonbuk 570-749, Republic of Korea

## Abstract

*Introduction*. Crotonis fructus (CF) is the mature fruit of *Croton tiglium* L. and has been used for the treatment of gastrointestinal disturbance in Asia. It is well known that the main component of CF is croton oil (CO). The present study is to investigate the effects of CF extracts (CFE) and CO on lipolysis in OP9 adipocytes. *Methods*. Glycerol release to the culture supernatants was used as a marker of adipocyte lipolysis. *Results*. Treatment with various concentrations of CFE and CO stimulates glycerol release in a dose-dependent manner. The increase in glycerol release by CFE is more potent than isoproterenol, which is a *β*-adrenergic agonist as a positive control in our system. The increased lipolysis by CFE and CO was accompanied by an increase of phosphorylated hormone sensitive lipase (pHSL) but not nonphosphorylated HSL protein and mRNA. Pretreatment with H89, which is a protein kinase A inhibitor, significantly abolished the CFE- and CO-induced glycerol release in OP9 adipocytes. These results suggest that CFE and CO may be a candidate for the development of a lipolysis-stimulating agent in adipocytes.

## 1. Introduction

*Croton tiglium L*. (family Euphorbiaceae) is a plant distributed in the tropical and subtropical zones of Asia. The fruit of* Croton tiglium L*. is Crotonis Fructus (CF), which is one of several medicines used for attenuating gastrointestinal diseases such as constipation, visceral pain, and intestinal inflammation [1, 2]. Additionally, the crude extracts of CF have been reported to activate M3 muscarinic receptor and Ca^2+^ influx through the L-type Ca^2+^ channel in isolated rabbit jejunum [3]. It is also well known that the main component of CF is the croton oil (CO). There is an abundance of linoleic acid, oleic acid, and eicosenoic acid in a methyl-esterified sample of CO [4]. It has been reported that CO may regulate gastrointestinal motility and induce intestinal inflammation related to immunological milieu and motor activity in mice [5].

Obesity is a medical condition in which excess body fat has accumulated to the extent that is associated with a variety of diseases, including metabolic syndrome and cardiovascular disease. White adipose tissue is the major fat reservoir in mammals storing the excess energy from the diet as triacylglycerol (TAG), a neural lipid composed of three fatty acids which is bound to the carbon backbone of a glycerol molecule [6]. One of the therapeutic methods for treating obesity is to reduce TAG in white adipocytes, which is termed lipolysis. Lipolysis is a complex process that is highly regulated and involves the coordinated participation of several lipases [7, 8]. Lipolysis occurs through the sequential hydrolysis of TAG by the action of three lipases: adipose triglyceride lipase (ATGL), hormone-sensitive lipase (HSL), and monoglyceride lipase (MGL) [9]. Among them, HSL is a key enzyme in the mobilization of fatty acids in adipocytes and its activity is regulated posttranscriptionally by reversible phosphorylation by protein kinase A (PKA) [10].

In this study, we used OP9 mouse stromal cells, first reported by Bickel and colleagues as a useful new model of adipocyte metabolism in 2006 [11]. In their study, OP9 cells initiated the same events, including lipid metabolism, insulin signaling, and glucose transport, much like 3T3-L1 cells [11]. We already reported about the inhibitory effects of* Pericarpium Zanthoxyli* extract on adipocyte differentiation by using OP9 cells [12].

In the present study, the effects of CF extract (CFE) and CO on lipolysis in OP9 cells were investigated by measuring glycerol release and evaluating HSL expression level.

## 2. Materials and Methods

### 2.1. Reagents

OP9 cells were purchased from the American Type Culture Collection (Manassas, VA, USA). Minimum essential medium alpha (MEM*α*) and fetal bovine serum (FBS) were purchased from Invitrogen (Carlsbad, CA, USA). Insulin, 3-isobutyl-1-methylxanthine (IBMX), dexamethasone (DEXA), isoguanosine, and croton oil (CO) were purchased from Sigma chemical (St. Louis, MO, USA). HA89, a protein kinase inhibitor, was purchased from Calbiochem (San Diego, CA). Antibodies against HSL and *β*-actin were purchased from Santa Cruz Biotechnology (Santa Cruz, CA, USA). Antibody against pHSL was obtained from Cell Signaling Technology (Beverly, MA, USA). All of the chemicals were of analytical grade.

### 2.2. Preparation of Crotonis Fructus Extracts (CFE)

Mature fruits of* Croton tinglium L*. were purchased from Kwangmyungdang Medicinal Herbs Co., Ltd. (Ulsan, Republic of Korea) and authenticated by Professor GS Lee, one of the authors. A voucher specimen (WKU030305-CT201305E) has been deposited at the Department of Herbology, College of Korean Medicine, Wonkwang University, Iksan, Korea.

The powdered Crotonis Fructus (100 g) was extracted using reflux method with 1000 mL of 70% aqueous ethanol for 2 hours. The extract was evaporated under 40 mmHg using a rotary evaporator and then freeze-dried. The yield of the final extract was 11.48% (w/w).

### 2.3. Cell Culture and Adipocyte Differentiation Induction

OP9 cells were cultured in MEM*α* containing 20% FBS, 2 mM l-glutamine, 100 U/mL penicillin, and 100 *μ*g/mL streptomycin at 37°C in a 5% CO_2_ incubator. To induce differentiation, 1-day postconfluent preadipocytes were incubated in a differentiation medium containing 10% FBS, 0.5 mM IBMX, 0.25 *μ*M DEXA, 175 nM insulin, 2 mM l-glutamine, 100 U/mL penicillin, and 100 *μ*g/mL streptomycin for 2 days. The medium was then changed to MEM*α* containing 10% FBS, 2 mM l-glutamine, and 175 nM insulin, and the cells were cultured for 3 days.

### 2.4. Determination of Cell Viability

The effect of CFE on OP9 cell viability was determined using an established MTT assay. The attached cells were kept untreated or treated with various concentrations of CFE (list the concentrations in parentheses) for 24 h at 37°C. The cells were washed with phosphate-buffered saline (PBS) prior to adding MTT (0.5 mg/mL PBS) and incubated at 37°C for 30 min. Formazan crystals were dissolved with dimethyl sulfoxide (100 *μ*L/well) and detected at OD_570_ with a model Emax (Molecular Devices, Sunnyvale, CA, USA).

### 2.5. Glycerol Release

After treatment of differentiated OP9 cells with various concentrations of CFE and CO, free glycerol content in cell supernatants was quantified using a glycerol quantification kit according to the manufacturer's instructions (Sigma Chemicals). Glycerol was quantified at OD540 with a model Emax (Molecular Devices). Isoproterenol (Merck, Darmstadt, Germany), a known stimulator of lipolysis, was used as a positive control.

### 2.6. Western Blot Analysis

The differentiated OP9 cells were pretreated with 20 *μ*M H89 for 2 h and then treated with 20 *μ*g/mL CFE and 10 *μ*g/mL CO for 12 h at 37°C. Cells were lysed with ice-cold M-PER mammalian protein extraction reagent (Pierce Biotechnology, Rockford, IL, USA), and the protein concentration in the lysate was determined using the Bradford method [13]. Samples (20 *μ*g) were separated by sodium dodecyl sulfate-polyacrylamide gel electrophoresis with 10% acrylamide and transferred to Hybond-P polyvinylidene fluoride (PVDF) membranes (GE Healthcare Life Sciences, Buckinghamshire, UK) using a western blot apparatus. Protein expression levels were determined by signal analysis using an image analyzer (Fuji-Film, Tokyo, Japan).

### 2.7. Quantitative Real-Time Polymerase Chain Reaction (PCR)

Total RNA was extracted from cells using a FastPure RNA kit (TaKaRa, Shiga, Japan). The RNA concentration and purity were determined by absorbance at 260/280 nm. cDNA was synthesized from 1 *μ*g of total RNA using a PrimeScript RT reagent kit (TaKaRa). Adipocyte differentiation-related gene mRNA expressions were determined by real-time PCR using the ABI PRISM 7900 Sequence Detection System and SYBR Green I (Applied Biosystems, Foster City, CA, USA). The forward and the reverse primers for HSL were 5′-GGAGCACTACAAACGCAACGA-3′ and 5′-TCGGCCACCGGTAAAGAG-3′, respectively; the primers for GAPDH were 5′-CGTCCCGTAGACAAAATGGT-3′ and 5′-TTGATGGCAACAATCTCCAC-3′, respectively. All results were normalized to the GAPDH housekeeping gene to control for variation in mRNA concentrations. Relative quantification was performed using the comparative ΔΔ*C*_*t*_ method according to the manufacturer's instructions.

### 2.8. Statistical Analysis

Statistical analysis was performed using OriginPro 8.0 software. One way analysis of variance (ANOVA) followed by Duncan's test. Data were expressed in means ± SD. Differences with *P* values less than 0.05 were considered statistically significant.

## 3. Results and Discussion

Crotonis Fructus (CF) is the mature fruit of* Croton tiglium L*. which has been used in traditional medicine for the treatment of gastrointestinal (GI) diseases including constipation, abdominal pain, peptic ulcer, and intestinal inflammation in Asia [2, 3, 5]. The main components of CF comprise the great parts of the extracted essential oil from seed and bark (fruit) of* Croton tiglium*, which is named by Croton tiglium oil (CO), and modulate intestinal transit in mice [5]. There were many reports about the effects of CF extracts and CO on GI problems [2, 3]. Kim et al. [14] also reported that isoguanosine from* Croton tiglium L.* has antitumor activity against implanted S-180 ascitic tumor in mice. However, there are no studies on their lipolytic effects on adipocytes, particularly on the underlying mechanism.

**Figure 1:**
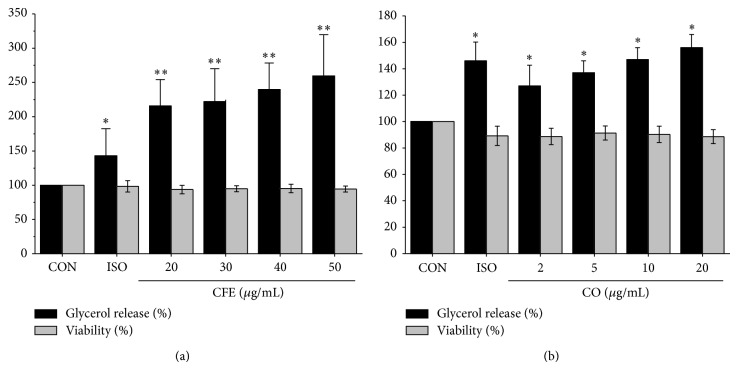
Effects of Crotonis Fructus extracts (CEF) and croton oil (CO) on glycerol release and viability. Differentiated OP9 adipocytes were treated with various concentrations of CFE (20–50 *μ*g/mL), CO (2–20 *μ*g/mL), and 200 nM isoproterenol (ISO) for 12 h. Glycerol release (black bar) was determined with a glycerol quantification kit and cell viability (gray bar) was quantified with MTT assay as described in Materials and Methods. Data are expressed as the mean ± SD of four independent experiments and a percentage of vehicle (DMSO-)treated control cells (CON). ^*^ *P* < 0.05, ^**^ *P* < 0.01 versus CON.

A significant increase in the amount of glycerol released into the media was observed in those OP9 adipocytes treated with CF extracts (CFE) (20–50 *μ*g/mL; *P* < 0.01) for 12 h (Figure 1(a)) in a dose-dependent manner. The increase in glycerol release by 20 *μ*g/mL CFE was observed after 6 h of treatment (1.6-fold, *P* < 0.05) and peaked after 12 h of treatment (2.2-fold, *P* < 0.01) (data not shown). Lipolysis of TAG into glycerol and fatty acids is activated by the *β*-adrenergic receptor agonist isoproterenol (ISO) and is used as a positive control in our system. ISO also increased glycerol release after 12 h of treatment (1.4-fold, *P* < 0.05). However, the effect of CFE on lipolysis is more potent than ISO, even at a concentration of 20 *μ*g/mL. We also checked the effects of CFE on ISO-induced lipolysis and the data revealed that CFE did not have any additional effect on the lipolytic effect of ISO (data not shown). And then, we want to know what the main constituent of CFE increasing glycerol release is. As shown in Figure 1(b), CO significantly increased glycerol release into the media at all concentrations tested, but it was less effective than CFE. We also tested the effects of isoguanosine on lipolysis and there was none (data not shown). We could not exclude the lipolytic effects of other compounds in CFE. None of CFE, CO, and isoguanosine at the concentration ranges tested was cytotoxic according to the MTT (Figure 1).

**Figure 2:**
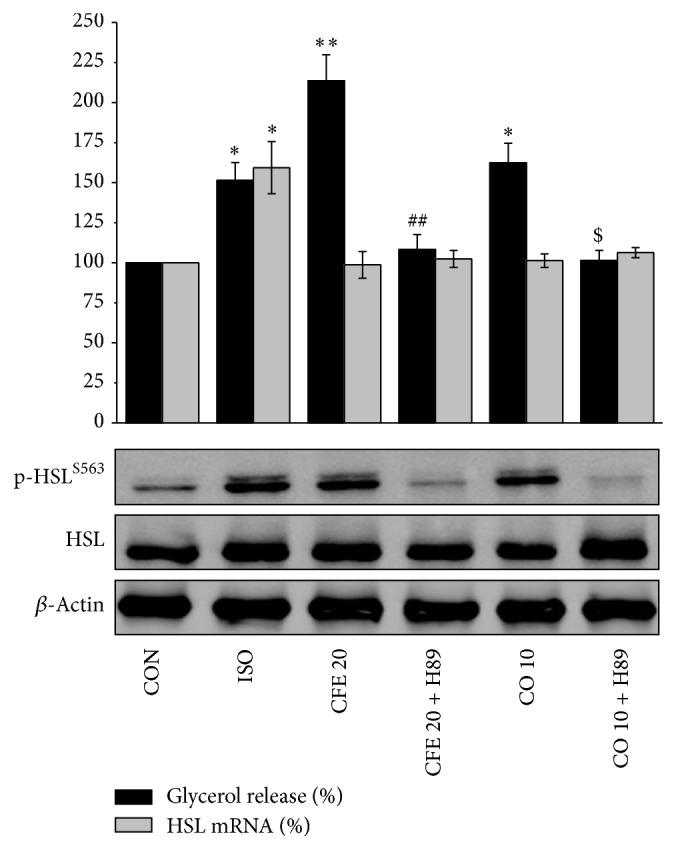
Signaling pathway involved in the lipolytic effects of CFE and CO. Differentiated OP9 adipocytes were pretreated with 20 *μ*M H89 and then treated with 20 *μ*g/mL of CFE, 10 *μ*g/mL of CO, and 200 nM ISO for 12 h. Glycerol release (black bar) quantification is the same as Figure 1 legends and real-time PCR for hormone sensitive lipase (HSL) mRNA (gray bar) was carried out using a specific primer for HSL as described in Materials and Methods. Protein expression levels for p-HSLS563 and HSL (lower panel) were subjected to western blot analysis by using specific antibodies. Data are expressed as the mean ± SD of four independent experiments and a percentage of vehicle (DMSO-)treated control cells (CON). ^*^ *P* < 0.05, ^**^ *P* < 0.01 versus CON; ^##^ *P* < 0.01 versus CFE-treated group; and ^$^ *P* < 0.05 versus CO-treated group.

In murine adipocytes, PKA phosphorylates HSL at several serine residues (563, 659, and 660), resulting in increased translocation of HSL to the lipid droplet surface and increased lipolytic activity [15]. To better elucidate the mechanisms underlying the lipolytic actions of CFE and CO, we investigated the effects of CFE and CO on HSL phosphorylation in Ser^563^. As shown in Figure 2, CFE (20 *μ*g/mL) and CO (10 *μ*g/mL) treatment for 12 h did not modify total protein and mRNA contents of HSL but significantly increased the phosphorylation of HSL at Ser^563^. However, the CFE- and CO-induced glycerol release and phosphorylation of HSL at Ser^563^ were significantly inhibited in the presence of the PKA inhibitor H89. These data suggest that CFE and CO stimulate lipolysis through HSL phosphorylation at Ser^563^ by PKA activation. In addition to the PKA-mediated phosphorylation, HSL may be phosphorylated by other kinases, such as extracellular signal-regulated kinase (ERK1/2), which activates HSL by phosphorylation [16]. Furthermore, AMP-activated protein kinase (AMPK) phosphorylates HSL at Ser^565^, which prevents phosphorylation induced PKA [17]. Other kinases such as ERK1/2 and AMPK may be involved in the underlying mechanism of CFE- and CO-induced glycerol release from lipid droplets.

Collectively, the present data demonstrate the ability of CFE to stimulate lipolysis in OP9 adipocytes, with CO as its major constituent. The lipolytic actions of CFE and CO are mainly mediated by the phosphorylation of HSL through PKA activation. These findings explain a mechanism by which CFE and CO induce lipolysis in adipocytes. This would further aid in the development of therapeutic strategy in preventing obesity.
